# B7H3 As a Promoter of Metastasis and Promising Therapeutic Target

**DOI:** 10.3389/fonc.2018.00264

**Published:** 2018-07-06

**Authors:** Peixin Dong, Ying Xiong, Junming Yue, Sharon J. B. Hanley, Hidemichi Watari

**Affiliations:** ^1^Department of Obstetrics and Gynecology, Hokkaido University School of Medicine, Hokkaido University, Sapporo, Japan; ^2^Department of Gynecology, State Key Laboratory of Oncology in South China, Sun Yat-sen University Cancer Center, Guangzhou, China; ^3^Department of Pathology and Laboratory Medicine, University of Tennessee Health Science Center, Memphis, TN, United States; ^4^Center for Cancer Research, University of Tennessee Health Science Center, Memphis, TN, United States

**Keywords:** B7H3, CD276, metastasis, epithelial-to-mesenchymal transition, cancer stem cells, microRNA

## Abstract

B7H3 (also known as CD276, an immune checkpoint molecule) is aberrantly overexpressed in many types of cancer, and such upregulation is generally associated with a poor clinical prognosis. Recent discoveries indicate a crucial role for B7H3 in promoting carcinogenesis and metastasis. This review will focus on the latest developments relating specifically to the oncogenic activity of B7H3 and will describe the upstream regulators and downstream effectors of B7H3 in cancer. Finally, we discuss the emerging roles of microRNAs (miRNAs) in inhibiting B7H3-mediated tumor promotion. Excellent recent studies have shed new light on the functions of B7H3 in cancer and identified B7H3 as a critical promoter of tumor cell proliferation, migration, invasion, epithelial-to-mesenchymal transition, cancer stemness, drug resistance, and the Warburg effect. Numerous miRNAs are reported to regulate the expression of B7H3. Our meta-analysis of miRNA database revealed that 17 common miRNAs potentially interact with B7H3 mRNA. The analysis of the TCGA ovarian cancer dataset indicated that low miR-187 and miR-489 expression was associated with poor prognosis. Future studies aimed at delineating the precise cellular and molecular mechanisms underpinning B7H3-mediated tumor promotion will provide further insights into the cell biology of tumor development. In addition, inhibition of B7H3 signaling, to be used alone or in combination with other treatments, will contribute to improvements in clinical practice and benefit cancer patients.

## Introduction

Metastasis, or the consequences of their treatment, are the primary cause of cancer death (1). Metastasis is commonly viewed as a multistep event resulting in the dissemination of tumor cells from the primary tumor site to a distant location (2). These include loss of gap junction and tight junction contacts with neighboring cells, migration and invasion of basement membrane and extracellular matrix, entry and survival in the blood vascular and lymphatic system, extravasation into the parenchyma of distant tissues, adaptation to tumor microenvironment and host tissue remodeling, and re-initiation of their proliferative programs at metastatic sites (3, 4).

Epithelial-to-mesenchymal transition (EMT) endows epithelial tumor cells with enhanced motility and invasiveness (5, 6). Furthermore, EMT-derived tumor cells acquire cancer stem cell (CSC) properties and exhibit therapeutic resistance (6–9). In addition, the mutual interactions between tumor cells and the surrounding tumor microenvironment will eventually promote tumor development and metastasis (10). Tumor microenvironment comprises many cell types including immune cells, fibroblasts, and endothelial cells (11). Tumor cells frequently display altered expression of cytokines and chemokines that promote the infiltration and activity of suppressive immune cell populations and also express immune checkpoint molecules (such as programmed cell death 1 ligand 1 and B7H3, also known as CD276) to inhibit the antitumor immune response (12–17).

B7H3 is expressed on immune cells (such as antigen-presenting cells or macrophages) and tumor cells and has inhibitory roles on T cells, contributing to tumor cell immune evasion (18–20). Recent studies have shown that B7H3 is a crucial player in tumor growth and metastasis beyond the immune regulatory roles (21). The developments in our understanding of cancer biology have provided a better understanding of how B7H3 regulates EMT and cancer stemness and of molecular mechanisms responsible for controlling the expression of B7H3 in cancer.

Although there have been substantial advances in our understanding of cancer at the molecular level, its prevention and treatment are still lacking. Considering the significant roles of B7H3 in cancer immunity and progression, the value of B7H3 in cancer diagnosis and treatment warrants further detailed study. Here, we review our current knowledge of how dysregulation of B7H3 and its signaling pathways can influence the hallmarks of cancer and discuss the potential use of microRNA (miRNA) as a potential therapeutic strategy for B7H3 overexpressing tumors, especially focusing on those miRNAs involved in the regulation of B7H3 expression in ovarian cancer.

## B7H3 Activation in Cancer

B7H3 (CD276) belongs to the B7 superfamily of immune checkpoint molecules (22). It is present at low levels in most normal tissues but is overexpressed in a wide variety of cancers, including bladder, breast, cervical, colorectal, esophageal, glioma, kidney, liver, lung, ovarian, pancreatic, prostate, intrahepatic cholangiocarcinoma, liver, oral squamous cell carcinoma, endometrial cancer, and squamous cell carcinoma and gastric cancer (23–42), glioma (43), and melanoma (44) (Table 1). Numerous studies showed that the overexpression of B7H3 was correlated with advanced tumor stage and high tumor grade in endometrial, cervical, breast, kidney cancer, and oral squamous cell carcinoma (25, 28, 30, 39, 40). The overexpression of B7H3 is associated with the proliferation and invasive potential of pancreatic, breast, colorectal, liver, prostate cancer, intrahepatic cholangiocarcinoma, and oral squamous cell carcinoma (26, 27, 30–32, 36–40). Notably, overexpression of B7H3 was found to correlate with poorer prognosis in many cancers (25, 28, 29, 31–34, 36–40, 44). However, high B7H3 expression predicts better survival for patients with gastric and pancreatic cancer (41, 45). A possible explanation for this discrepancy could be different cancer type (or subtypes), tumor heterogeneity, differences in sample size, and clinical stage, the time point of B7H3 measurement and the different methodology used in research.

**Table 1:** The association between B7H3 expression and clinicopathologic factors of human cancers.

Cancer type	No.	Method	Expression	Clinical factors					Reference
					
				Size	Stage/grade	Invasion depth	LN meta/recurrence	Survival	
Bladder, breast, cervical, colorectal, esophageal, kidney, liver, lung, ovarian, pancreatic, prostate cancer, glioma, melanoma	1,342	IHC	Upregulation	NA	NA	NA	NA	NA	(23)
Bladder cancer	302	IHC	Upregulation	−	−	−	−	NA	(24)
Endometrial cancer	107	IHC	Upregulation	−	+	NA	NA	Poor	(25)
Pancreatic cancer	26	ELISA	Upregulation	+	NA	NA	NA	NA	(26)
Pancreatic cancer	59	IHC	Upregulation	NA	+	NA	+	NA	(27)
Cervical cancer	108	IHC	Upregulation	+	−	−	−	Poor	(28)
Breast cancer	90	IHC	Upregulation	−	−	−	−	Poor	(29)
Breast cancer	82	IHC/qPCR	Upregulation	+	+	−	+	NA	(30)
Intrahepatic cholangiocarcinoma	45	IHC	Upregulation	−	−	+	+	Poor	(31)
Colorectal cancer	275	IHC	Upregulation	NA	+	+	−	Poor	(32)
Ovarian cancer	103	IHC	Upregulation	NA	+	−	−	Poor	(33)
Glioma	41	IHC/microarray	Upregulation	NA	+	−	−	NA	(43)
Melanoma	97	IHC/qPCR	Upregulation	NA	+	−	−	Poor	(44)
Lung cancer	270	IHC	Upregulation	NA	+	NA	NA	Poor	(34)
Lung cancer	70	IHC	Upregulation	NA	−	NA	+	NA	(35)
Liver cancer	24	IHC	Upregulation	NA	+	+	−	Poor	(36)
Prostate cancer	823	IHC	Upregulation	NA	NA	+	+	Poor	(37)
Prostate cancer	2,111	Microarray	Upregulation	NA	+	NA	+	Poor	(38)
Oral squamous cell carcinoma	NA	IHC	Upregulation	+	+	−	−	Poor	(39)
Kidney cancer	743	IHC	Upregulation	+	+	NA	NA	Poor	(40)
Pancreatic cancer	96	IHC/qPCR	Upregulation	NA	−	−	−	Better	(41)
Gastric cancer	32	IHC/qPCR	Upregulation	−	−	−	−	Better	(42)

*LN meta, lymph node metastasis; NA, data were not available*.

**Figure 1:**
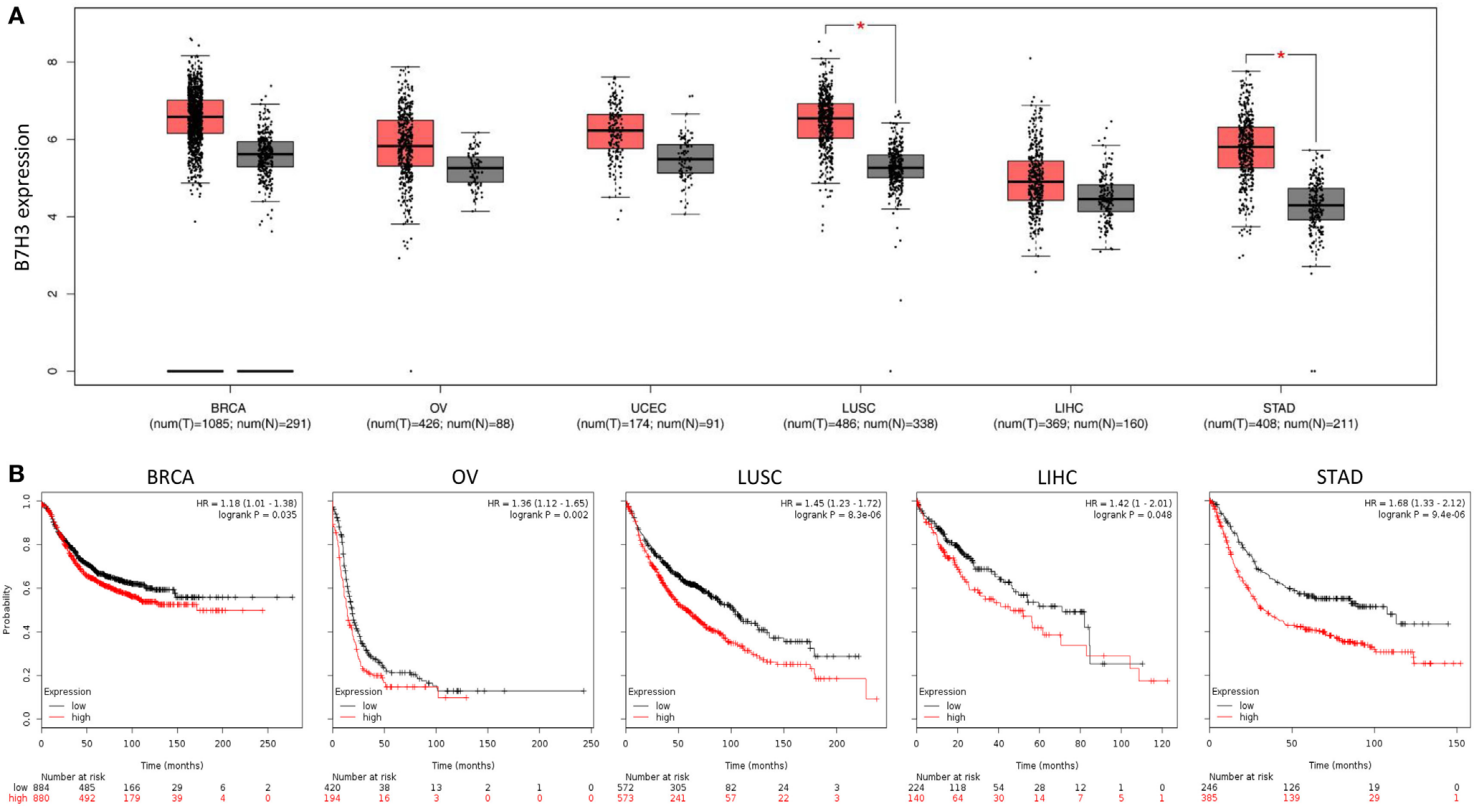
High expression of B7H3 was correlated with poorer prognosis in cancers. **(A)** B7H3 expression profile across TCGA pan-cancer datasets. Images were taken from the GEPIA (Gene Expression Profiling Interactive Analysis) online database (http://gepia.cancer-pku.cn). N, normal; C, cancer. *P < 0.05. **(B)** Kaplan–Meier curves for overall survival in indicated cancer types using the Kaplan–Meier Plotter database (www.kmplot.com). Red and black lines indicate patients with higher and lower than median *B7H3* mRNA expression, respectively. High expression of *B7H3* was significantly correlated with shorter overall survival in each Kaplan–Meier plotter cohort. BRCA, breast invasive carcinoma; OV, ovarian serous cystadenocarcinoma; UCEC, uterine corpus endometrial carcinoma; LUSC, lung squamous cell carcinoma; LIHC, liver hepatocellular carcinoma; STAD, stomach adenocarcinoma.

We assessed B7H3 expression in TCGA pan-cancer datasets obtained from Gene Expression Profiling Interactive Analysis (GEPIA) online database.^1^ In agreement with previous reports, RNA sequencing analysis of mRNA expression from the GEPIA online database (46) revealed that B7H3 expression levels tend to be higher in breast, ovarian, endometrial, lung, liver, and gastric cancer tissues compared to corresponding normal tissues (Figure 1A). We also characterized the association between B7H3 mRNA expression and prognosis in several cancers using the Kaplan–Meier plotter database^2^ (47). Higher expression of B7H3 was significantly associated with shorter overall survival in breast, ovarian, lung, liver, and gastric cancer (Figure 1B).

## The Roles of B7H3 in Different Cancer Cells and Possible Mechanisms

The following sections and Table 2 summarize the current understanding of the functional role of B7H3 in metastasis and describe its underlying mechanisms in different tumor cells.

**Table 2:** Roles, functions, and mechanisms of B7H3 in cancer.

Cancer type	Role	Function	Mechanism	Reference
Prostate cancer	Oncogene	Migration, invasion	NA	(48)
Melanoma/breast cancer	Oncogene	Migration, invasion	NA	(49)
Melanoma/breast cancer	Oncogene	Migration, invasion	Increased the expression of MMP2, STAT3, and IL-8	(50)
Melanoma	Oncogene	Proliferation, glycolytic capacity, resistance to chemotherapy and small-molecule inhibitors	NA	(51)
Breast cancer	Oncogene	Paclitaxel resistance	Activated JAK2/STAT3 pathway	(64)
Breast cancer	Oncogene	Glucose uptake, lactate production, proliferation	Increased the expression of HIF1α and its downstream targets, LDHA and PDK1	(70)
Gastric cancer	Oncogene	Migration, invasion, proliferation	NA	(42)
Gastric cancer	Oncogene	Migration, invasion	Increased CXCR4; and activated AKT, ERK, and JAK2/STAT3 phosphorylation	(52)
Esophageal squamous cell carcinoma	Oncogene	Migration, invasion	NA	(53)
Liver cancer	Oncogene	Proliferation, adhesion, migration, and invasion	NA	(54)
Pancreatic cancer	Oncogene	Proliferation, invasion	NA	(26)
Colorectal cancer	Oncogene	Resistance to chemotherapy	Activated JAK2/STAT3 pathway	(65)
Colorectal cancer	Oncogene	Oxaliplatin resistance	Increased the expression of XRCC1 *via* PI3K/AKT pathway	(66)
Colorectal cancer	Oncogene	Migration, invasion	Activated JAK2/STAT3/MMP9 pathway	(55)
Colorectal cancer	Oncogene	Resistance to chemotherapy	Increased BRCC3 expression	(67)
Colorectal cancer	Oncogene	Resistance to chemotherapy	Activated PI3K/AKT/TS pathway	(68)
Colorectal cancer	Oncogene	Epithelial-to-mesenchymal transition, cancer stemness	Decreased E-cadherin expression and increased of N-cadherin, Vimentin, CD133, CD44, and OCT4 expression	(59)
Osteosarcoma	Oncogene	Invasion	Increased the expression of MMP2	(56)
Pancreatic cancer	Oncogene	Invasion, metastasis	Activated TLR4/NF-κB signaling and increased IL-8 and VEGF expression	(57)
Glioma	Oncogene	Migration, invasion, cancer stemness	NA	(61)
Ovarian cancer	Oncogene	Resistance to chemotherapy and small-molecule inhibitors, cancer stemness	Possibly increased the expression of ALDH	(60)

*NA, data were not available*.

## Roles of B7H3 in Cancer Cell Proliferation and Invasiveness

Evidence supporting a tumor-promoting role for B7H3 is now increasingly apparent from functional studies of diverse malignancies. A lot of evidence demonstrated that B7H3 is involved in biological processes of cancer development, such as proliferation, migration, and invasion. For instance, knockdown of B7H3 expression in prostate, breast, gastric, liver, pancreatic, colorectal cancer cells, and melanoma cells could significantly suppress cell migration and invasion (26, 42, 48–57).

Different molecular mechanisms may also underlie these effects: (1) B7H3 induced the migratory potential and invasiveness of tumor cells by increasing the expression of metastasis-associated proteins such as MMP2, STAT3 and IL-8 (50); (2) by increasing the levels of CXCR4 and activating AKT, ERK, and JAK2/STAT3 pathways (52); (3) through activating the JAK2/STAT3/MMP9 pathway (55); (4) by increasing the expression of MMP2 (56); (5) by activating the TLR4/NF-κB signaling and increased IL-8 and VEGF expression (57).

Several studies have provided convincing *in vivo* functional data that are consistent with the data from cancer cell lines and thus support the tumor-promoting role of B7H3 during cancer progression. For example, in the subcutaneous transplantation pancreatic cancer mouse model, tumor growth rate was reduced by the knockdown of B7H3 (26). Similarly, the silencing of B7H3 significantly decreased tumor proliferation in mantle cell lymphoma *in vitro* and *in vivo* (58).

## B7H3 Mediates EMT and CSC in Cancer Cells

Some researchers claimed that B7H3 plays a key role in modulating EMT and CSC-like properties of various cancer cells. B7H3 can promote EMT and cancer stemness by decreasing E-cadherin expression and increasing the expression of N-cadherin, Vimentin, CD133, CD44, and OCT4 (59). Blockade of B7H3 with a monoclonal antibody reduced the number of cancer-initiating cells (60). A previous study found that B7H3 is an inducer of cell invasion and sphere formation in glioma cells (61), further suggesting a role of B7H3 in the cancer invasion process.

Cancer stem cells or tumor-initiating cells not only possess the ability of self-renewal but also develop strong resistance to chemotherapy (62). It was demonstrated that the induction of EMT generated cells with properties of CSCs (63). In breast cancer and colorectal cancer cells, B7H3 induced the resistance to paclitaxel or 5-fluorouracil (5-FU) through activating the JAK2/STAT3 pathway (64, 65). In addition, a few other mechanisms may also underlie B7H3-mediated chemoresistance: (1) B7H3 induces oxaliplatin resistance by increasing the expression of XRCC1 *via* PI3K/AKT pathway (66); (2) B7H3 also enhances cell resistance to chemotherapy by increasing the expression of BRCC3, which antagonizes DNA damage caused by 5-FU (67); (3) or *via* the activation of the PI3K/AKT pathway (68).

## Role of B7H3 in Cancer Metabolism

Warburg effect (or aerobic glycolysis) is a metabolic hallmark of cancer, characterized by an excessive conversion of glucose to lactate even with ample oxygen (69). A recent study found that B7H3 can promote the Warburg effect, evidenced by increased glucose uptake and lactate production in breast cancer cells. Furthermore, this stimulating effect of B7H3 on the Warburg effect was also observed in a mouse model of breast cancer (70). Mechanistically, B7H3-induced metabolic shift in cancer cells is mediated by HIF1α, a master regulator in the reprogramming of cancer metabolism in favor of glycolysis (70), revealing a new mechanism for the Warburg effect in cancer cells. Reasonably, we believe treating tumors by targeting their metabolism through modulation of B7H3 expression would probably generate a better effect of tumor eradication.

## Regulatory Mechanisms of B7H3 in Cancer

Protein expression is usually controlled by the following mechanisms: the genetic aberrations of the gene loci (71), transcriptional regulation (72), posttranscriptional regulation at the mRNA level (73), and protein modification (74). Epigenetic mechanisms such as DNA methylation (75), histone modification (76), and non-coding RNAs (77, 78) play a key role in regulating gene expression. DNA methylation and modification of histones mediate gene transcription, and miRNAs regulate gene expression posttranscriptionally (79). To date, it is less clear whether B7H3 overexpression observed in cancer is due to genomic DNA amplification, or which transcription factors are responsible for B7H3 transcription. However, chromatin immunoprecipitation analysis in prostate cancer cells revealed an androgen receptor-binding site upstream of B7H3, and the presence of androgens decreased B7H3 expression (38).

Interestingly, immunoglobulin-like transcript-4 (ILT4) is an inhibitory receptor that inhibits the function of certain immune cells and was shown to upregulate B7H3 expression *via* the PI3K/AKT/mTOR signaling in lung cancer cells (80). Co-expression of ILT4 and B7H3 was positively corelated with lymph node metastasis and advanced tumor stage (80). Consequently, further study is needed to elaborate the link between ILT4 and B7H3 in different cancer cells.

At the posttranscriptional level, numerous miRNAs, including miR-214, miR-363*, miR-326, miR-940, miR-29c, miR-665, miR-34b*, miR-708, miR-601, miR-124a, miR-380-5p, miR-885-3p, and miR-593, directly interact with the 3′-UTR of B7H3 mRNA, resulting in attenuation of B7H3 expression in breast cancer (81). miR-124 also binds directly to the 3′-UTR of B7H3 mRNA, inhibiting its expression in osteosarcoma (82). TGF-β1 through SMAD3 and SMAD4 elevated miR-155 expression, which in turn attenuated CEBPB expression and consequently miR-143 expression in colorectal cancer cells. As a result, the reduction of miR-143 led to the upregulation of B7H3, a direct target of miR-143 (83). These results indicated that TGF-β1 may promote cancer immune escape by upregulating B7H3 expression. In addition, a recent study demonstrated that p53 binds to the promoter of miR-124 to elevate its expression in colorectal cancer cells (84). Meanwhile, iASPP, a novel oncoprotein overexpressed in many cancers, interacts with p53 to suppress p53-mediated transcription of target genes (75, 85). Thus, these results indicate a possible mechanism underlying B7H3 overexpression in tumors: iASPP-mediated p53 repression leads to the downregulation of miR-124, subsequently resulting in increased expression of B7H3.

**Figure 2:**
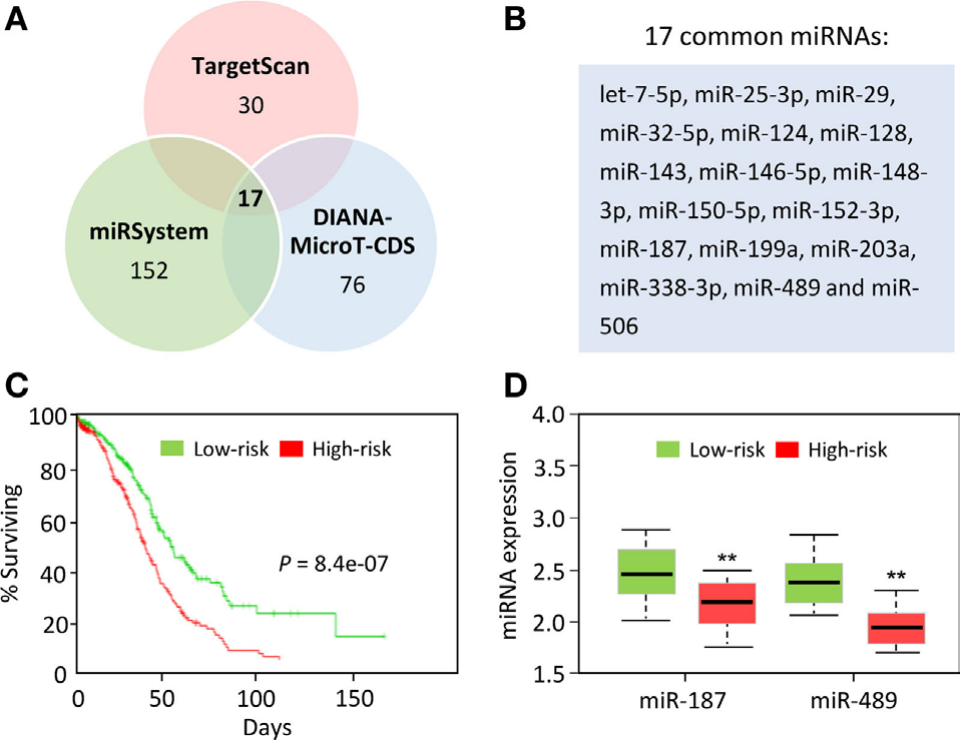
MicroRNAs (miRNAs) that potentially regulate B7H3 expression in ovarian cancer. **(A)** Venn diagram showing the overlap of miRNAs that were predicted to bind to the *B7H3* 3′-UTR by alternative algorithms (TargetScan, miRSystem, and DIANA-MicroT-CDS). **(B)** The 17 predicted miRNAs were common to these three algorithms. **(C)** The Kaplan–Meier survival curves of 458 TCGA (Cancer Genome Atlas database) ovarian cancer samples were created using the SurvMicro database based on the low (*n* = 229) or high (*n* = 229) risk for a poor outcome. **(D)** Box plots demonstrating significantly lower levels of miR-187 and miR-489 expression in the high-risk ovarian cancer patients.

We used three computational algorithms, including TargetScan,^3^ miRSystem,^4^ and DIANA-MicroT-CDS^5^ to identify miRNAs that might regulate B7H3 expression. This analysis revealed 17 common miRNAs predicted to bind the 3′-UTR of the B7H3 transcript (Figures 2A,B). In colorectal cancer cells, a recent study showed that miR-187 binds B7H3 mRNA and suppresses its expression to inhibit cell proliferation, migration, invasion, and induced cell apoptosis (86). In clear cell renal cell carcinoma, another study confirmed that B7H3 expression is downregulated by miR-187, a tumor suppressor that suppresses cancer cell proliferation and motility (87). Collectively, these data suggest that the loss of tumor suppressor miRNAs activate B7H3 and contributes to cancer progression.

We further evaluated the correlation of patient survival with the expression of these miRNAs in ovarian cancer samples in the TCGA by using the online software SurvMicro.^6^ Ovarian patients were stratified into the high-risk (with a low probability of survival; *n* = 229) or low-risk (with a high probability of survival; *n* = 229) group (*P* = 8.4E−07, Figure 2C). High-risk patients had lower miR-187 and miR-489 expression levels than the low-risk patients (Figure 2D). Thus, these 17 miRNAs, especially miR-187 and miR-489, are expected to have binding sites in the 3′-UTR of B7H3 in cancer cells, although functional validation remains to be performed.

## Conclusion

Interruption of metastasis pathways holds preclinical and clinical promise as an anti-metastasis therapy. The emerging role of B7H3 in human tumor cells and in inducing EMT/CSC-like features have been noted. Furthermore, tumor cells could rely on Warburg effect to generate energy (88). The recent findings led to the identification of B7HH3 as a contributor to the Warburg effect (70). Therefore, targeting the metastatic potential and metabolic changes with inhibitors against B7H3 may be a promising way for cancer therapy.

The induced B7H3 expression has been detected in multiple cancers as compared with normal tissues. The B7H3 protein, especially when located in the cell membrane, may be a perfect choice for targeted drug development. Importantly, the treatment with an inhibitory B7H3 monoclonal antibody in melanoma cells leads to decreased proliferation and Warburg effect (51). Additionally, targeting B7H3 with a monoclonal antibody has demonstrated the safety and efficacy in the salvage treatment of stage IV childhood neuroblastoma (43). Activated T cell (ATC) armed with a novel anti-CD3 × anti-B7H3 bispecific antibody was found to significantly inhibit lung cancer growth *in vivo* compared with unarmed ATC (89), indicating that targeting B7H3 represent a novel alternative to improve current cancer therapy.

Future studies aimed at delineating the precise cellular and molecular mechanisms underpinning B7H3-mediated tumor promotion will provide further insights into the cell biology of tumor development. In addition, inhibition of B7H3 signaling, to be used alone or in combination with other treatments, will contribute to improvements in clinical practice and benefit cancer patients.

## Author Contributions

PD and HW provided direction. PD, YX, and HW wrote the manuscript. JY and SH made significant revisions to the manuscript. All authors read and approved the final manuscript.

## Conflict of Interest Statement

The authors declare that the research was conducted in the absence of any commercial or financial relationships that could be construed as a potential conflict of interest.

## Footnotes

^1^http://gepia.cancer-pku.cn (Accessed: June 5, 2018).

^2^http://kmplot.com/analysis/ (Accessed: June 5, 2018).

^3^http://www.targetscan.org/vert_72/ (Accessed: June 5, 2018).

^4^http://mirsystem.cgm.ntu.edu.tw/ (Accessed: June 5, 2018).

^5^http://diana.imis.athena-innovation.gr/DianaTools/index.php?r=microT_CDS/index (Accessed: June 5, 2018).

^6^http://bioinformatica.mty.itesm.mx:8080/Biomatec/Survmicro.jsp (Accessed: June 5, 2018).
